# Bone Regeneration of Hydroxyapatite/Alumina Bilayered Scaffold with 3 mm Passage-Like Medullary Canal in Canine Tibia Model

**DOI:** 10.1155/2015/235108

**Published:** 2015-01-26

**Authors:** Jong Min Kim, Jun Sik Son, Seong Soo Kang, Gonhyung Kim, Seok Hwa Choi

**Affiliations:** ^1^Xenotransplantation Research Center, Department of Microbiology and Immunology, Institute of Endemic Diseases, College of Medicine, Seoul National University, Seoul 110-799, Republic of Korea; ^2^Korea Textile Development Institute, Daegu 703-702, Republic of Korea; ^3^Department of Veterinary Surgery, Chonnam National University, Gwangju 500-757, Republic of Korea; ^4^Veterinary Medical Center, Chungbuk National University, Cheongju 361-763, Republic of Korea

## Abstract

The aim of this study was to evaluate the bone regeneration of hydroxyapatite (HA)/alumina bilayered scaffold with a 3 mm passage-like medullary canal in a beagle tibia model. A porous HA/alumina scaffold was fabricated using a polymeric template-coating technique. HA/alumina scaffold dimensions were 10 mm in outer diameter, 20 mm in length, and with either a 3 mm passage or no passage. A 20 mm segmental defect was induced using an oscillating saw through the diaphysis of the beagle tibia. The defects of six beagles were filled with HA/alumina bilayered scaffolds with a 3 mm passage or without. The segmental defect was fixated using one bone plate and six screws. Bone regeneration within the HA/alumina scaffolds was observed at eight weeks after implantation. The evaluation of bone regeneration within the scaffolds after implantation in a beagle tibia was performed using radiography, computerized tomography (CT), micro-CT, and fluorescence microscopy. New bone successfully formed in the tibia defects treated with 3 mm passage HA/alumina scaffolds compared to without-passage HA/alumina scaffolds. It was concluded that the HA/alumina bilayered scaffold with 3 mm passage-like medullary canal was instrumental in inducing host-scaffold engraftment of the defect as well as distributing the newly formed bone throughout the scaffold at 8 weeks after implantation.

## 1. Introduction

Bone defects often occur as a result of trauma, bone tumors, resection, and metabolic diseases. Bone lesions above a critical size are hard to cure. Most cases lead to nonunion. To treat long bone defects, numerous biomaterials were developed. Among them, porous hydroxyapatite (HA) is widely used because of its good bone healing outcomes [1]. Calcium phosphate-based bioceramics such as HA possess excellent biocompatibility because its composition is similar to the apatite found in natural bone [2, 3]. HA has been commonly used for the fabrication of highly porous, interconnected scaffolds and isotropic pore structures. Therefore, HA ceramics have been used extensively as a substitute in bone grafts [4, 5]. Since the 1980s, blocks and granules of porous calcium HA ceramics have been used in orthopaedic, dental, or craniofacial surgery [6]. A canine defect model showed successful bone formation, mineralization, and vascularization when the defects were filled with highly porous and interconnected HA scaffolds [7, 8]. Our previous study in a large segmental defect model showed successful new bone formation when the defects were filled with highly porous and interconnected HA scaffolds [9]. Although the porous HA scaffolds previously fabricated have good open channels, the effectiveness of a scaffold with a passage-like medullary canal is unknown for applications in large segmental defects.

The aim of this study was to evaluate the bone regeneration of HA/alumina bilayered scaffold with 3 mm passage-like medullary canal, comparing scaffolds with and without passages through radiography, computerized tomography (CT), micro-CT, and fluorescence microscopy in a beagle tibia model.

## 2. Materials and Methods

### 2.1. Porous HA/Alumina Bilayered Scaffold

**Figure 1:**
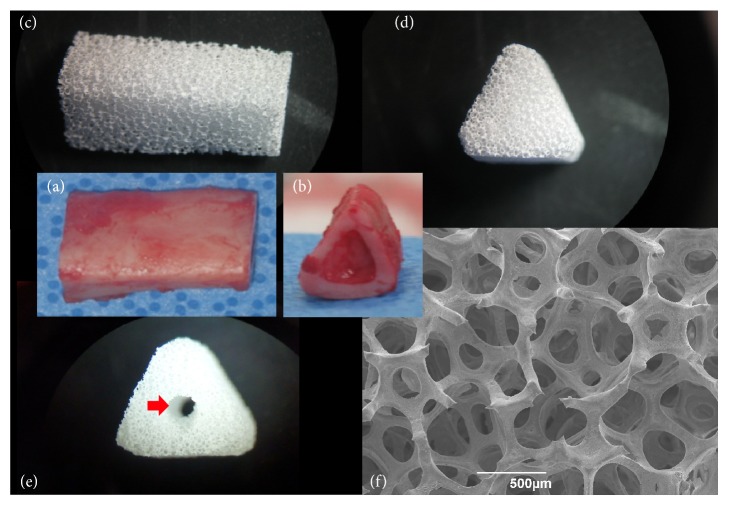
Various images of HA/alumina bilayered scaffolds. (a) and (b) Lateral and frontal photographs of 20 mm resected tibia in a beagle. (c)–(e) Stereoscope images of a scaffold. (c) Lateral image of a scaffold. (d) Front image of without passage in a scaffold. (e) Front image with 3 mm passage (arrow) in a scaffold. (f) SEM image of a scaffold.

Porous HA/alumina scaffold was fabricated using a polymeric template-coating technique as previously described [3]. A polyurethane sponge with 60 pores per inch (E.N. Murray Co., Denver, USA) was coated with nanoalumina powders (OssGen Co., Daegu, Korea) in distilled water-based slurry. Binders (3% high molecular weight polyvinyl alcohol, 3% carboxy-methylcellulose, 5% ammonium polyacrylate dispersant, and 7%* N,N-*dimethylformamide drying agent) were added to the slurry mixture to improve sintering and stabilize the scaffold structure. Coated sponges were dried overnight at room temperature before sintering at 1500°C for 3 h. The alumina scaffolds were coated with HA slurry in the same manner as above and resintered at 1230°C for 3 h. Final HA/alumina scaffold dimensions were 10 mm in outer diameter, 20 mm in length, and with either a 3 mm passage or no passage (Figure 1). The properties of the scaffold produced were observed using a stereoscope (Fisher Micromaster, Fisher Scientific, USA) and a scanning electron microscope (SEM; EVO 40, ZEISS, USA).

### 2.2. Surgical Procedures

A total of six adult beagle dogs in healthy condition, aged two years old with an average weight of 8.2 kg, were used in this study. Under general anesthesia, aseptic surgical preparation of the left tibia was performed. Skin, muscle, and periosteum were incised over the medial side of tibia for exposure of tibia bone. A 20 mm segmental defect per a dog was induced using oscillating saw in the diaphysis of tibia. As a tested group, the defects of four dogs were filled with HA/alumina bilayered scaffolds with 3 mm passage-like medullary canal, whereas the defects in the other two dogs were filled scaffolds without passage. The fracture was fixated using one stainless bone plate and six screws, followed by suturing. The experimental protocol was approved by the Institutional Animal Care and Use Committee of Chungbuk National University, Korea.

### 2.3. Radiographic, CT, and Micro-CT Observations

Radiographic images were taken under general anesthesia at 0, 2, 4, 6, and 8 weeks after implantation to assess the new bone formation and mineralization. Radiographs were obtained using an X-ray machine (Rotanode, Toshiba, Japan) from a distance of 100 cm, at 60 kVp and 300 mA, and with an exposure time of 0.03 s. CT images were obtained by a single-slice spiral CT (Hi Speed CT/e, GE Medical Co., USA) at eight weeks after implantation after removal of a bone plate and screws at autopsy. CT exposures were performed at 120 kVp (130 mA), with 1 mm thickness and 512 × 512 voxel matrix. Three-dimensional (3D) image software (mimics 13.1, Materialise Co., Belgium) was used to process the CT images. Micro-CT was used to develop 3D images of tibial bone with HA/Alumina in defects at eight weeks following implantation. A Skyscan Desktop micro-CT 1172 (Aartselaar, Belgium), with a source voltage of 60 kV, a current of 167 *μ*A, and resolution of 26.7 *μ*m, was used to acquire X-ray radiographs. The specimens were attached to a stage that rotated 180° with images acquired every 0.6°. After scanning, cross-sectional slices were reconstructed and each scan result was reconstructed using the 0.008~0.031 threshold values to distinguish bone and air. The reconstructions of scanned images were performed using NRecon software (Skyscan Aartselaar, Belgium). The 3D images were obtained through 3D Vol software (Skyscan Aartselaar, Belgium).

### 2.4. Fluorescent Bone Labeling and Preparation of Specimens for Fluorescence Microscopy

Fluorescent labels were administered to the beagles to monitor new bone formation around scaffold. Calcein (Sigma-Aldrich Co, USA, 10 mg/kg, IV) was administered at six weeks after implantation. Alizarin red S (Sigma-Aldrich Co, USA, 30 mg/kg, IP) was administered at eight weeks after implantation at three days before autopsy. The formalin-fixed samples from the sacrificed defect area in each beagle were dehydrated in a graded series of ethanol and then immersed and infiltrated using Technovit 7200VLC before light catalysis for 24 h. A macrocutting and grinding system (Exakt 310 CP series; Exakt Apparatebau, Norderstedt, Germany) was used to produce undecalcified cut and ground sections. A final thickness of approximately 50 *μ*m of each section was produced for fluorescence microscopy. Labeling of new bone formation was evaluated with confocal laser scanning microscope. Active bone formation was evaluated relative to the presence or absence, intensity, and width of the fluorochrome markers.

## 3. Results and Discussion

### 3.1. Materials Characterization

HA scaffolds were fabricated using the polymeric sponge replication method, which has received particular attention because it can provide very high porosity with good interconnections between the pores. The stereoscopic image of HA/alumina bilayered scaffold is shown in Figures 1(c), 1(d), and 1(e). The scaffold was triangular in structure, similar to the beagle tibial bone (Figures 1(a) and 1(b)). As shown in Figure 1(e), SEM indicated scaffold architecture with open pores and interconnected rod-like struts. Open channels were observed to be arranged with isotropic geometry and rounded-edge triangular strut morphology. The pore sizes ranged from 230 *μ*m to 470 *μ*m, and porosity was 91.61 ± 1.28%. Alumina is suited for biomaterial application due to a high degree of chemical inertness, excellent wear resistance, ability to be polished to a high surface finish, and excellent hardness [10]. Therefore, the combined application of HA and alumina to bilayered scaffolds in this study is a good strategy to support mechanical strength for repairing large segmental bone defects.

### 3.2. General Observation, Radiographic, CT, and Micro-CT Analyses

**Figure 2:**
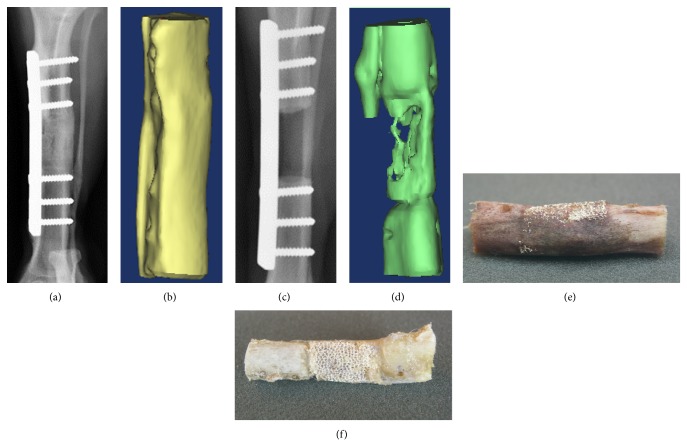
Various images of beagle tibiae with scaffold in defaced sites (20 mm) at 8 weeks following implantation. (a), (b), and (e) Segmental defect site with 3 mm passage in a scaffold. (c), (d), and (f) Segmental defect site without passage in a scaffold. (a) and (c) Radiographs of segmental defect sites. (b) and (d) 3D CT reconstructed images of segmental defect sites after removal of plate and screws at autopsy. (e) and (f) Gross images of segmental defect sites.

The bone regenerated to bridge the defect and formed new cortex in the region with the 3 mm passage as shown in Figure 2(e) but not in those without a passage as shown in Figure 2(f). Scaffolds with a 3 mm passage exhibited better integration with the host bone on the proximal and distal ends of the defect compared to those without a passage. The ceramic portion of those with a 3 mm passage was not identifiable in the region except for the plate. However, all ceramic portions were identifiable in the region without a passage. By eight weeks after implantation, perfect bony union was observed between the scaffold with the 3 mm passage and the host tissues. To evaluate bone healing and the development of bone union within the defects, radiographic images were taken at eight weeks following implantation (Figures 2(a) and 2(c)). Without the passage where defects were left unfilled, no bone formation was observed in the defects during the entire experimental period. The extremities of the bone defects were sclerotic, and the medullary cavities were blocked at eight weeks after implantation, suggesting that the bone defect was not repaired. However, in defects that were filled with scaffolds with a 3 mm passage, new bone formation was observed at the interfacial area between the scaffold and the host bone at eight weeks after implantation, suggesting union at the proximal and distal host bone-implant interfaces. Callus formation was detected at eight weeks after implantation around the periphery of the scaffold with a 3 mm passage as well as along the adjacent host bone, indicating the formation of mineralized tissue in the pores of the scaffold of 3 mm passage. CT 3D-image showed that the original bone contour was almost fully restored in the 3 mm passage and that the scaffold-filled defect site was completely healed at eight weeks after implantation but not in the defects without passage (Figures 2(b) and 2(d)). Some research did not observe callus formation at any time during the 16 weeks in dogs when 21 mm defect of the canine femoral diaphysis was treated with HA/TCP scaffolds of 60% porosity [11]. This result is similar to our without a passage. In contrast, our 3 mm passage shows the segmental bone defect in the tibia was archived new bone formation at eight weeks after implantation. This observation suggests that the 3 mm passage-like medullary canal is important for the blood circulation, improving bone regeneration.

**Figure 3:**
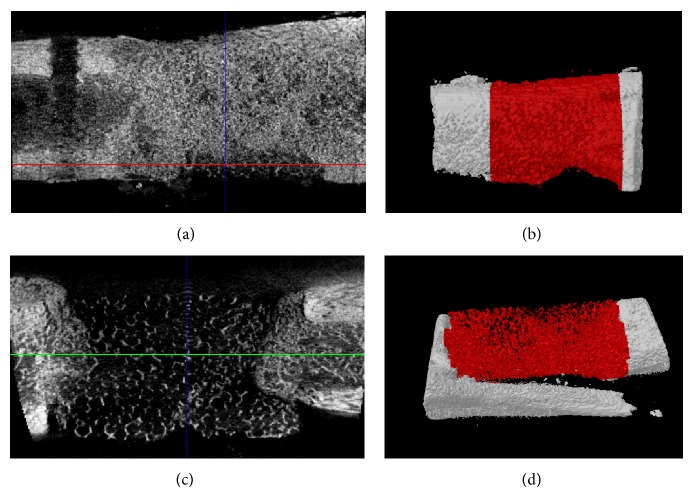
Micro-CT images of segmental defect sites treated with a 3 mm passage ((a) and (b)) or without passage ((c) and (d)) scaffold in beagle tibiae at eight weeks after implantation. (a) and (c) Sagittal images. (b) and (d) 3D micro-CT reconstructed images.

Figure 3 shows micro-CT sagittal images and 3D reconstructed images of the defect with scaffold either with a 3 mm passage or without a passage at eight weeks after implantation. New bone formation in the open-scaffold pores, with complete healing, was observed when the defect site was filled with scaffolds with 3 mm passages in sagittal image but not in the without passages. According to the 3D reconstructed images (Figures 3(b) and 3(d)), dense bone regenerated in the scaffolds, with the scaffold-bone interface becoming indistinguishable in those with 3 mm passages, but not in those without. This observation suggested successful engraftment of the scaffolds to bone in the 3 mm passages. Yuan et al. reported that unions of scaffold in segmental defect were made at the host bone and implant junctions, whereas the middle portion of these scaffolds showed fibrous tissue deposition [12]. Interestingly, in those with 3 mm passages in this study, the union at host-bone-implant interface was observed, with bone formation occurring throughout the scaffolds by eight weeks after implantation but not in those without a passage. Observations from this study therefore suggest that the HA/alumina bilayered scaffold with a 3 mm passage is suitable for osteogenesis and is applicable as an ideal scaffold for repair of large segmental bone defects.

### 3.3. Fluorescence Microscopic Evaluation

**Figure 4:**
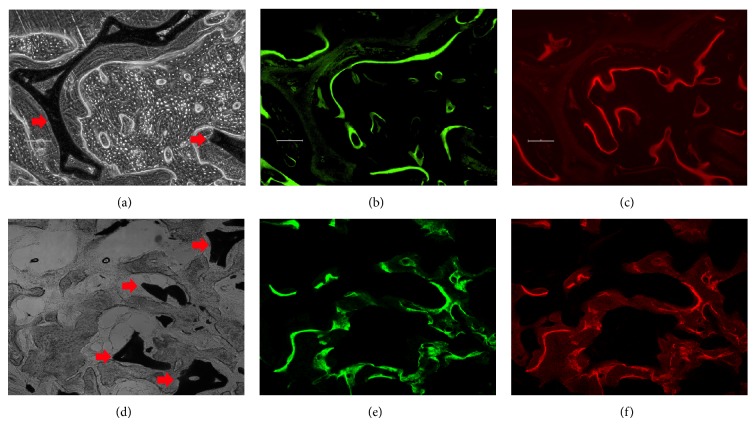
Fluorescence microscopic images of segmental defect sites treated with 3 mm passage ((a)–(c)) or without passage ((d)–(f)) scaffold in beagle tibiae at eight weeks after implantation. (a) and (d) nonstained images. (b) and (e) Green color (calcein) indicates new bone formation at six weeks after implantation on the confocal microscopy. (c) and (f) Red color (alizarin red S) indicates new bone formation at eight weeks following implantation on the confocal microscopy. Arrows indicate a scaffold.

The 3 mm passages showed very new bone formation around and along the scaffold, whereas it was clearly reduced in the without passages in nonstained images (Figures 4(a) and 4(d)). The obtained images showed areas labeled in green and red that represented regions of calcium precipitation labeled by fluorochromes at different moments of tissue mineralization. The calcein labeling (green) represents the regions where calcium precipitated at six weeks after implantation. The alizarin labeling (red) represents the regions where calcium precipitated at eight weeks after implantation. Most of the substances applied at six weeks after implantation were closest to the scaffold surface in all scaffolds, and the substances administered at eight weeks following implantation were found further away from the scaffold surface (Figures 4(b), 4(c), 4(e), and 4(f)). There are many reports showing good bone regeneration in* in vivo* studies using porous scaffold [13, 14]. However, to the best of authors' knowledge, no studies have been conducted to determine whether a scaffold with a passage-like medullary canal can result in faster new bone formation.

## 4. Conclusion 

In this study, the* in vivo *performance of porous HA/alumina bilayered scaffolds with isotropic pore structures either with a 3 mm passage or without a passage was evaluated in 20 mm segmental bone defects of beagle tibiae. The scaffold with a 3 mm passage-like medullary canal demonstrated that new bone was better formed in large segmental defects compared to the scaffold without a passage. A passage-like medullary canal in a scaffold appears important for the blood circulation to improve the rate of bone regeneration.
